# Magnetic Resonance Imaging with Superparamagnetic Iron Oxide Fails to Track the Long-term Fate of Mesenchymal Stem Cells Transplanted into Heart

**DOI:** 10.1038/srep09058

**Published:** 2015-03-12

**Authors:** Ning Ma, Huaibing Cheng, Minjie Lu, Qiong Liu, Xiuyu Chen, Gang Yin, Hao Zhu, Lianfeng Zhang, Xianmin Meng, Yue Tang, Shihua Zhao

**Affiliations:** 1 Department of Radiology, State Key Laboratory of Cardiovascular Disease, Fuwai Hospital, National Center for Cardiovascular Diseases, Chinese Academy of Medical Sciences and Peking Union Medical College; 2 Comparative Medical Center, Peking Union Medical College (PUMC) and Institute of Laboratory Animal Science, Chinese Academy of Medical Science (CAMS); 3 Core Laboratory, State Key Laboratory of Cardiovascular Disease, Fuwai Hospital, National Center for Cardiovascular Diseases, Chinese Academy of Medical Sciences and Peking Union Medical College; 4 Experimental Animal Center, State Key Laboratory of Cardiovascular Disease, Fuwai Hospital, National Center for Cardiovascular Diseases, Chinese Academy of Medical Sciences and Peking Union Medical College

## Abstract

MRI for in vivo stem cell tracking remains controversial. Here we tested the hypothesis that MRI can track the long-term fate of the superparamagnetic iron oxide (SPIO) nanoparticles labelled mesenchymal stem cells (MSCs) following intramyocardially injection in AMI rats. MSCs (1 × 10^6^) from male rats doubly labeled with SPIO and DAPI were injected 2 weeks after myocardial infarction. The control group received cell-free media injection. In vivo serial MRI was performed at 24 hours before cell delivery (baseline), 3 days, 1, 2, and 4 weeks after cell delivery, respectively. Serial follow-up MRI demonstrated large persistent intramyocardial signal-voids representing SPIO during the follow-up of 4 weeks, and MSCs did not moderate the left ventricular dysfunction. The TUNEL analysis confirmed that MSCs engrafted underwent apoptosis. The histopathological studies revealed that the site of cell injection was infiltrated by inflammatory cells progressively and the iron-positive cells were macrophages identified by CD68 staining, but very few or no DAPI-positive stem cells at 4 weeks after cells transplantation. The presence of engrafted cells was confirmed by real-time PCR, which showed that the amount of Y-chromosome-specific SRY gene was consistent with the results. MRI may not reliably track the long-term fate of SPIO-labeled MSCs engraftment in heart.

In the past decades, myocardial infarction (MI) remains a leading cause of death and disability although advances in risk factor modification, drug treatment, and revascularization therapy have significantly reduced the mortality. Stem cell-based cell therapy is currently emerging as a promising treatment for patients with MI^1^. Bone-marrow-derived MSCs are easily obtained and expanded multipotent progenitor cells^2^, and have been the focus of numerous preclinical studies and clinical trials^3^,^4^,^5^,^6^,^7^,^8^. Prior studies have reported that MSCs injection improved myocardial contractile function, ventricular remodeling, and myocardial perfusion^9^. However, there is still numerous unresolved and controversial issues remain to be answered, more specifically, with regard to the underlying mechanisms for the functional benefits. Therefore tracking the implanted cells in vivo such as temporal changes of cell location, viability and functional status is of great clinical importance. MRI is a promising modality for in vivo stem cell tracking because it offers morphological and functional imaging with a high spatial resolution. Studies have reported that MRI can track the engrafted MSCs' fate in vivo after these cells were labeled with SPIO, and SPIO do not affect cell viability, proliferation, differentiation or migration^10^,^11^. However, other studies have raised several concerns, such as the specificity of MRI imaging to the presence of cells^12^,^13^,^14^,^15^. Namely, the hypointense signal is maintained by SPIO at a site regardless of cell viability and SPIO are present not necessarily within implanted cells at longer time points^12^, but rather in phagocytosing monocytes following engrafted stem cells death^13^. Winter et al. reported the absence of any discrimination between healthy successfully engrafted stem cells and dead stem cells phagocytosed by macrophages within the heart. In particular, no differences in signal voids up to more than 40 days were observed with dead and viable cells recipient with respect to size, number and localization^14^. Similarly, it has been demonstrated that MRI overestimates the SPIO labelled stem cells survival after transplantation in the heart^15^. Thus, the aim of this study was to determine whether MRI can track the long-term fate of the SPIO nanoparticles labeled adult rat MSCs including survival and migration in rat models of myocardial infarction following intramyocardially injection.

## Methods

### Animals

The animal experiments were approved by the Institutional Animal Care and Use Committee at FuWai hospital and the Beijing Council on Animal Care including any relevant details, and all experiments were performed in accordance with the “Guide for the care and use of laboratory animals” published by the US National Institutes of Health(publication no. 85-23, revised 1996). All animals received humane care. Lewis rats were obtained from Beijing WTLH Experimental Animal Corporation (Beijing, China; certificate no. SCXK2004-2005 [Beijing]).

### Isolation and culture of rat MSCs and cell labeling

Isolation and culture of rat MSCs were performed as previously described^16^,^17^,^18^,^19^. The surface antigen profiles and the potential for multi-lineage differentiation were analyzed in previous study^16^. Briefly, MSCs were harvested from femurs and tibias of 3-week-old male Lewis rats (60–80 g). The cells were seeded in Iscove's modified Dulbecco's medium (IMDM) culture medium (Gibco) with L-glutamine supplemented with 10% fetal bovine serum (FBS, Gibco) and 100 U/mL each of penicillin and streptomycin on flasks. Culture media was replaced after 2 days and changed twice a week thereafter. Cells of the hematopoietic lineage and other non-adherent cells were washed off during medium changes. The cells were dissociated with 0.25% trypsin and 1 mM ethylenediaminetetraacetic acid (EDTA, Sigma) and replated to expand the cells through successive passages. For cell labeling, MSCs were grown in complete culture medium containing 25 mg/mL SPIO (Feridex IV, Advanced Magnetics) and 0.375/mL poly-L-lysine (Sigma) in a humidified 5% CO_2_ incubator at 37°C for 48 h and followed by washing three times in phosphate-buffered saline (PBS) to rinse off SPIO particles on the surface of cells. The uptake of iron by cells was examined by Prussian blue staining and microscopic examination. Cells were resuspended accordingly in fresh complete medium with DAPI (Sigma) in a humidified 5% CO_2_ incubator at 37°C for 15 min and followed by washing four times in PBS to rinse off DAPI before explantation. The viability of the cells before transfer was assessed by trypan-blue exclusion. The transplanted MSCs were passage 3 and 4.

### Creation of the rat myocardial infarction model and cell transfer

MI was induced as previously described^20^. Simply, 8-week-old female Lewis rats (weighing 190–200 g) were anesthetized with intraperitoneal chloral hydrate, intubated and mechanically ventilated with room air at 70 breaths/min using a small animal ventilation apparatus (model 683; Harvard Apparatus). The heart was exposed via anterior thoracotomy, and the left anterior descending coronary artery was permanently ligated a few millimeters from its origin with an intramural stitch (6-0 Prolene suture). Successful performance of coronary occlusion was confirmed by myocardial blanching in the distal myocardium. The thorax was then closed with three layers of sutures. Postoperatively, rats were placed in a cage on a warming pad, supplied with oxygen, and monitored for several hours before being transferred back to the animal colony. Two weeks after MI, the chest was reopened and rats were randomized to receive 1 × 10^6^ SPIO-labeled MSCs (in ≈25 μL saline) or PBS by direct injection into the peri-infarcted area at 1 site for each heart.

### MRI

MRI was performed on the 2 groups using a 7.0 T horizontal-bore animal scanner(Varian) supplied with an actively-shielded gradient of 400 mT/m and a 70-mm transmit/receive birdcage radio frequency (RF) coil, at 24 hours before cell delivery (baseline), 3 days, 1, 2, and 4 weeks after cell delivery, respectively. Imaging sequences used for post-implantation MSCs localization and heart function included electrocardiographic (ECG) gating cine images and a T2*-weighted gradient echo sequence, which is a highly sensitive sequence to detect the susceptibility artifacts (hypointensities) generated by the SPIO-labeled cells. Animals were anesthetized with isoflurane (3% for induction and 1.5–2% for maintenance) in oxygen. Small animal ECG electrodes (SA Instruments), attached to the two forelimbs, and a respiration-detection cushion placed under the thorax, were used for monitoring of and triggering on ECG and respiration signals. The depth of anesthesia was continuously regulated to maintain a stable respiration rate during the experiment. Body temperature was maintained at 35°C. After three-dimensional plane localization, two-chamber views, four-chamber views and short-axis views were acquired. A stack of contiguous 1 mm short-axis ECG-gated gradient echo cine-MRIs were acquired to cover the entire left ventricle using the following parameters: repetition time (TR) = 10 ms, echo time (TE) = 1.8 ms, field-of-view (FOV) = 40 × 40 mm, matrix size 196 × 196, flip angle 20°, slice thickness 1 mm, 4 averages, 10 cardiac phases. Regions of interests (ROIs) were selected within hypointensitive area and normal myocardium in the slice of maximum hypointensities (T2* signal area) of T2*-weighted images. The signal intensity (SI) and hypointensitive area was estimated from such ROIs using ImageJ (NIH, Bethesda, MD). The signal contrast ratio was expressed as a percentage and calculated as:

Signal contrast ratio = (SI_normal myocardium_ − SI_hypointensitive area_)/SI_normal myocardium_*100%

### Histological analysis and immunohistochemistry

For histology, 2 animals from each of the 2 groups was killed after MRI scans at 3 days, 1 week, 2 weeks, and 4 weeks after cell transplantation, respectively. Hearts were harvested and fixed in 10% formaldehyde solution. Conventional paraffin-embedding and serial sections of MSCs engrafted area were carried out. Hematoxylin and eosin (HE) staining and Prussian blue staining were then performed, respectively. To identify the cells that had taken up the iron particles, we performed antibody staining for the macrophage marker CD68 (Sigma) in sections consecutive to those stained with Prussian blue. Primary antibody was incubated overnight. As secondary antibody, biotinylated goat anti rabbit (Sigma) was used. Staining was visualized using the horseradish peroxidase reaction with 3,3′-diaminobenzidine tetrahydrochloride (DAB).

### TUNEL analysis

Heart tissue of MSCs engrafted area was evaluated using the TUNEL assay to identify MSCs undergoing apoptosis at 3 days, 1 week, 2 weeks, and 4 weeks after cell transplantation, respectively. Portions of the heart were fixed with 10% paraformaldehyde. 6-micrometer thick frozen sections were prepared and stained by the TUNEL method using an in situ apoptosis detection kit (Roche Diagnostics) according to the manufacturer's instructions. Each stained section was examined at high power fields (200×). The number of TUNEL positive transplanted MSC was counted and the percentage of TUNEL positive cells was calculated per field using the following formula: (TUNEL positive nuclei/total DAPI-stained nuclei) ×100%. Five fields of each section were randomly selected.

### Measurement of SRY gene by real time PCR

RT-PCR analysis for the rat Y-chromosome-specific SRY gene was performed to detect the survival of the implanted cells after MRI scans at 3 days, 1 week, 2 weeks, and 4 weeks after cell transplantation, respectively. Genomic DNA was extracted from the sample and used for SRY detection. The primers for rat SRY gene were forward 5′-TGAACAGAATGGAACGGAGCA-3′ and reverse 5′-TGCTGCCTTGTATGGGAGC-3′; those used for β-actin were forward 5′-TCAATGACAACTTTGTCAAGCTCA-3′ and reverse 5′-GTGGGTGGTCCAGGGTTTCTTACT-3′.Quantitative real-time PCR was performed using a standard SYBR Green detection by the ABI Prism 7700 sequence detector (Applied Biosystems). The results were finally calculated by 2^−ΔΔCt^ method.

### Statistical analysis

Results were presented as mean ± standard deviation(SD). Statistical comparisons were made using two-way analysis of variance (ANOVA), two-way repeated measures ANOVA, unpaired Student's t-tests or non-parametric equivalents as appropriate. P value < 0.05 was considered statistically significant. Data were analyzed using SPSS 16.0 software (version 16.0).

## Results

### Myocardial infarction operation and mortality

**Figure 1:**
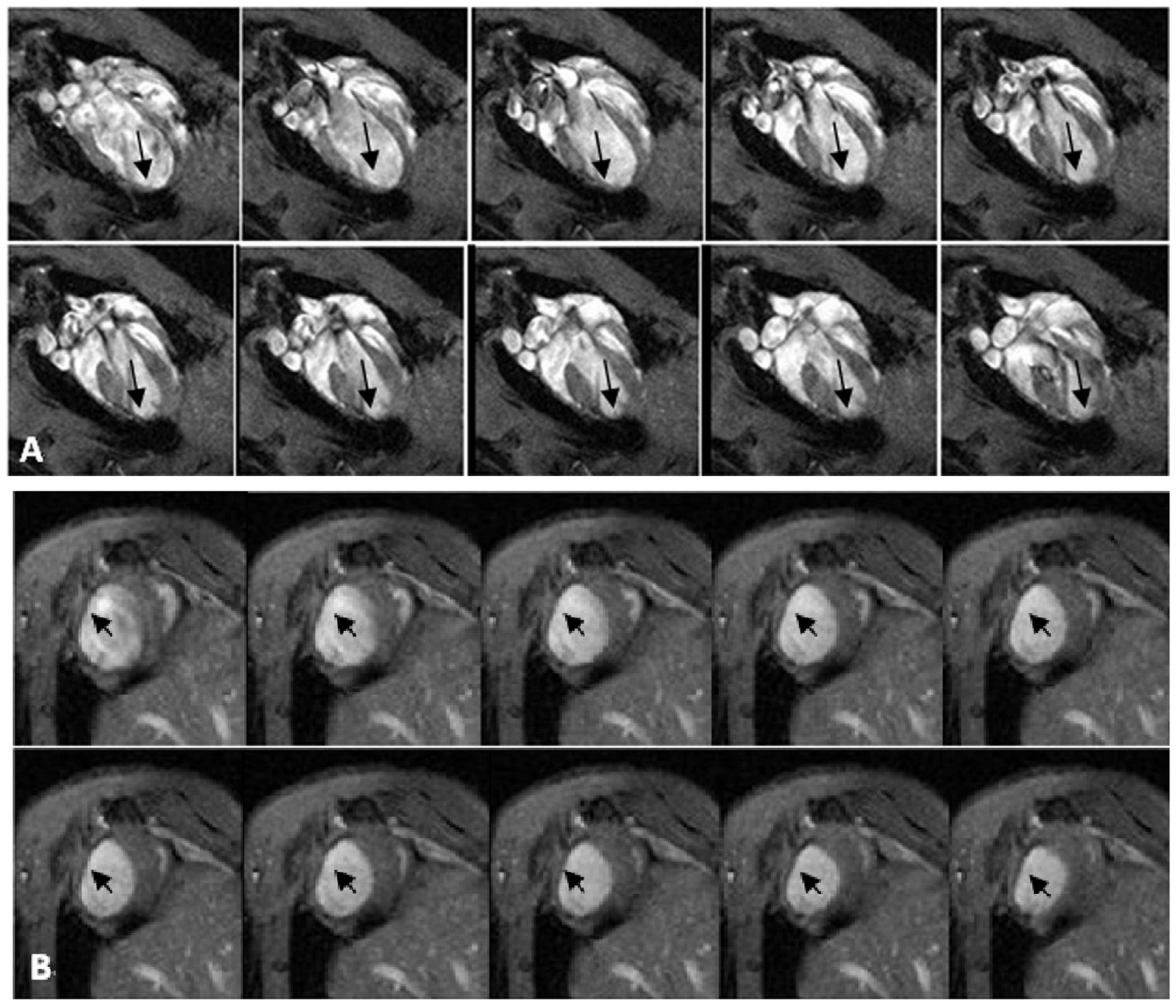
Cine MR imaging performed 2 weeks after MI. The four-chamber views (A) and short-axis views (B) showed that the anterior wall of left ventricle became thinner, the motion of the anterior and apical wall decreased or disappeared, and aneurysm appeared after myocardial infarction.

**Figure 2:**
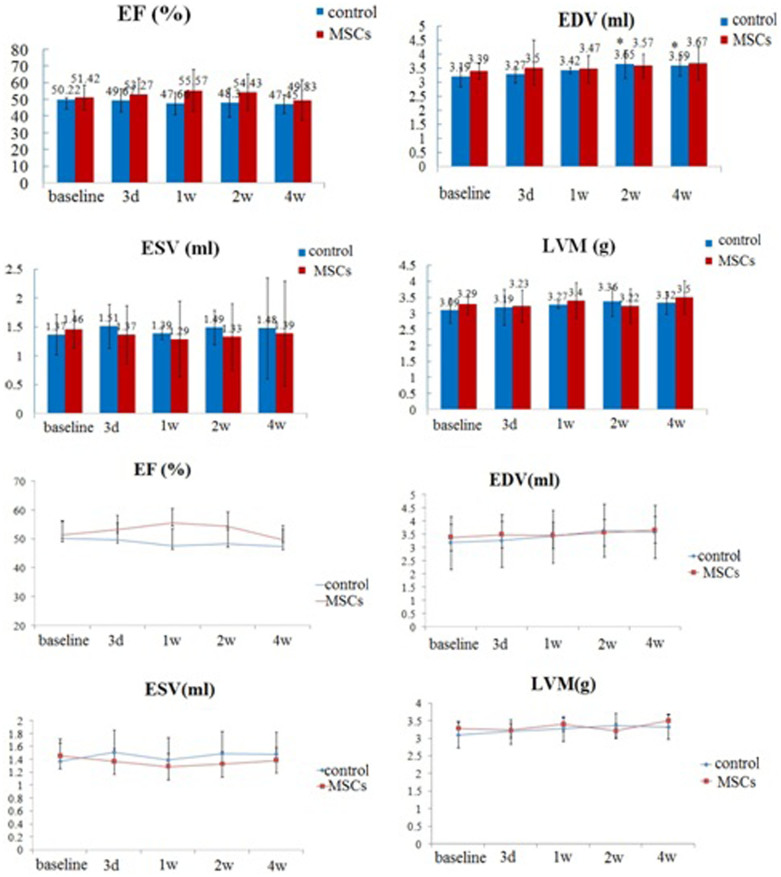
Comparison of LV function estimated by serial MRI. Compared with baseline and the controls, left ventricular ejection fraction did not increased significantly and there were no statistical differences in left ventricular end-systolic volume, * indicates P < 0.05, The end-diastolic volume increased in control group at 2 weeks and 4 weeks after transplantation when compared to baseline. N = 7,8,8,7 for MSCs group at 3 days, 1 w, 2 w, 4 w after MSCs injection respectively, n = 6,6,7,7 for control group at 3 days, 1 w, 2 w, 4 w after PBS injection respectively. LVEF: left ventricular ejection fraction; EDV: left ventricular end-diastolic volume; ESV: left ventricular end-systolic volume; LVM: left ventricular mass.

Overall, 110 rats were used in the study, and 27 died after the ligation of the left anterior descending branch (26% postoperative mortality). 8 rats were found to have no or only very small infarctions on MRI and were not included in the study. And apnea death occurs in 5 rats during the MRI scan. Thus, the remaining qualified animals(see Figure 1) were randomly divided into two groups: (1) MI without MSCs engrafted(control group, n = 35); (2) MI with MSCs engrafted(MSCs group, n = 35). The differences of the cardiac function parameters among the two groups of animals were not statistically significant (baseline EF: control 50.22 ± 5.74 vs MSCs 51.42 ± 7.41, P > 0.05, Figure 2). A total of 14 rats died during cell injection, 5 in the MSCs group and 9 in the control group.

### MSCs culture and labeling

**Figure 3:**
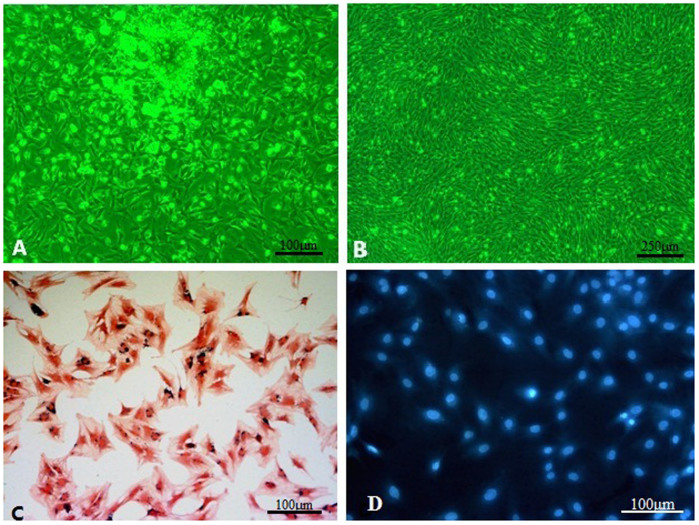
(A&B) Morphology of the MSCs under the inverted microscope. (A) The phenotype of the cells at passage 0 was heterogeneous (round shaped, irregular shaped and spindle shaped cells) forming different cell colonies. (original magnification, 100×). (B) The phenotype of the passage 3 cells was homogeneous spindle shaped cells at 80–90% confluence (original magnification, 40×). (C) Eosin and Prussian blue staining of SPIO-labeled MSCs. The labeling efficiency of MSCs using SPIO and PLL reached 90%. Clustered blue particles were present in most of the cells (original magnification, 100×). (D) DAPI-labeled MSCs under the inverted fluorescence microscope. DAPI-labeled MSCs at passage 3 observed by fluorescence microscopy (original magnification, 100×).

At 24 h after inoculation, adherent spindle- and rod-shaped cells were visible, and a large number of red blood cells were suspended in the culture medium (Figure 3A). Five to six days after inoculation, the cells had reached 80% confluence and were arranged in radial and gyrate patterns. MSCs were purified with increased passaging. The MSCs showed a typical fibroblast-like morphology by passage 3 (Figure 3B).

The labeling efficiency of MSCs labeled with SPIO at a concentration of 25 μg Fe/ml reached 90%. Clustered blue particles were present in most of the cells (Figure 3C). Almost 100% MSCs showed bright blue intranuclear fluorescence by fluorescence microscopy after 15 min labeling (Figure 3D).

### Left ventricular function estimated by in vivo MRI

Magnetic resonance examination was performed in 2 groups of rats with MI at baseline (1 day before cell transplantation) and 3 days, 1 week, 2 weeks and 4 weeks later. The typical course of LV wall thinning, LV dilatation and functional deterioration was observed in 2 groups. The left ventricular ejection fraction (LVEF, %), left ventricular end-diastolic volume (EDV), left ventricular end-systolic volume (ESV), left ventricular mass (LVM) were assessed respectively (Figure 2). Cardiac function was not restored significantly at 3 days, 1 week, 2 weeks and 4 weeks after MSCs transplantation compared with baseline (53.27 ± 9.44 vs 51.42 ± 7.41; 55.57 ± 12.52 vs 51.42 ± 7.41; 54.43 ± 10.99 vs 51.42 ± 7.41; 49.83 ± 12.17 vs 51.42 ± 7.41; P > 0.05, Figure 2). Compared with the controls, left ventricular ejection fraction did not increase significantly (49.67 ± 7.03 vs 53.27 ± 9.44; 47.66 ± 6.76 vs 55.57 ± 12.52; 48.30 ± 8.61 vs 54.43 ± 10.99; 47.45 ± 5.54 vs 49.83 ± 12.17; P > 0.05, Figure 2) and there were no statistical differences in left ventricular end-systolic volume (1.51 ± 0.38 vs 1.37 ± 0.57; 1.39 ± 0.11 vs 1.29 ± 0.66; 1.49 ± 0.30 vs 1.33 ± 0.58; 1.48 ± 0.87 vs 1.39 ± 0.91; P > 0.05, Figure 2). The end-diastolic volume increased in control group at 2 weeks and 4 weeks after transplantation when compared to baseline (3.65 ± 0.50 vs 3.19 ± 0.35; 3.59 ± 0.36 vs 3.19 ± 0.35; P < 0.05, Figure 2).

### Stem cell tracking in animals by MRI

**Figure 4:**
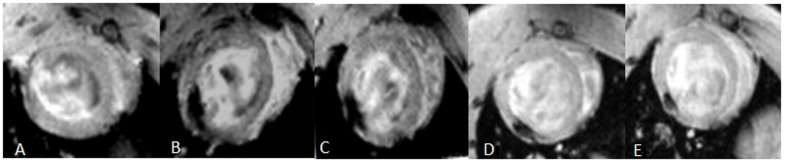
Serial MRI. (A) Control saline-injected heart had no hypointensities. (B) Injection of 1 × 10^6^ SPIO-labeled MSCs 3 days created a wide intramural area of hypointensity (arrows) at the anterior LV wall. (C–E) Positive magnetic signals (arrows) are still visible at 1 w, 2 w, 4 w after MSCs injection respectively, but the signal intensity changed gradually.

**Figure 5:**
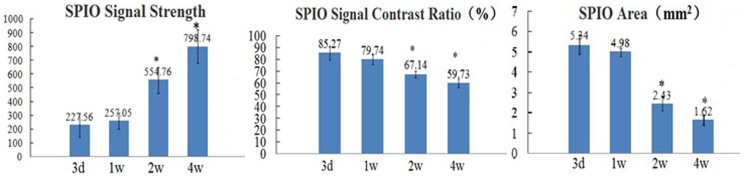
Comparison of SPIO detected by serial MRI. The SPIO signal strength, the signal contrast ratio (%) and the low signal area compared with the normal myocardium at different time points after cell transplantation. * indicates P < 0.05, the MSCs group at 2 and 4 weeks after cell transplantation compared with 3 days after transplantation. The signal intensity of normal myocardium which we detected was 1797.27 ± 231.38. N = 7,8,8,7 for MSCs group at 3 days, 1 w, 2 w, 4 w after MSCs injection respectively.

We performed serial MRI exams to track the cells at 3 days, 1, 2, and 4 weeks after cell injection into the peri-infarct zone of the LV anterior wall. Well-defined hypointensities (“black spots”) were observed at the region of cell injection in all the animals who received SPIO-labeled MSCs (Figure 4B–E). In the sagittal section of the LV 3 days after treatment, the site of injection demonstrated wide hypointensities which extended beyond the whole thickness of the LV wall, while the actual area of the labeled cells was smaller, which was called blooming effect. Control saline-injected hearts had no hypointensities on the MRI (Figure 4A). Four weeks after cell delivery, “black spots” could still be visualized by MRI (Figure 4E). The signal strength of hypointensities increased, the area of hypointensities decreased gradually and the boundary blurred (Figure 4B–E). There were no statistical differences in the signal strength, the area of “black spots” and the signal contrast ratio(%) compared with the normal myocardium between 1 week and 3 days after MSCs injection (227.56 ± 87.62 vs 257.05 ± 56.43; 5.34 ± 0.45 vs 4.98 ± 0.22; 85.27 ± 6.04 vs 79.74 ± 4.41, P > 0.05, figure 5). But the signal strength was significantly higher, the low signal area and signal contrast ratio(%) also decreased significantly at 2 and 4 weeks after the transplantation of MSCs (figure 5). Compared with 3 days after transplantation, the low signal area, the signal strength and signal contrast ratio(%) were significantly different at 2 and 4 weeks (554.76 ± 95.25 vs 227.56 ± 87.62; 798.74 ± 121.89 vs 227.56 ± 87.62; 5.34 ± 0.45 vs 2.43 ± 0.34; 5.34 ± 0.45 vs 1.62 ± 0.23; 85.27 ± 6.04 vs 67.14 ± 2.67; 85.27 ± 6.04 vs 59.73 ± 3.58; P < 0.05, figure 5).

### Distribution and apoptosis of transplanted stem cells detected by fluorescence microscopy

**Figure 6:**
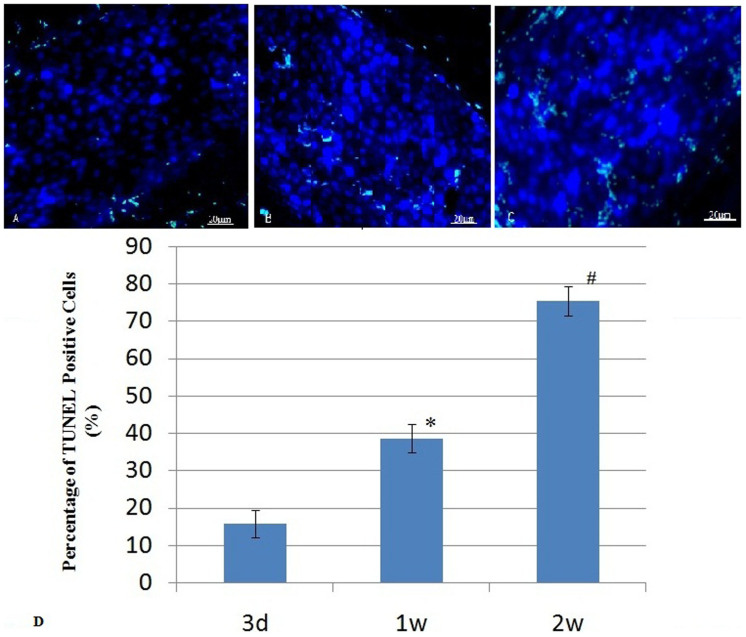
TUNEL analysis of heart tissue sample at the site of MSCs injection. (A) Fluoromicrograph showed many MSCs engrafted (blue nuclei) in infarcted hearts after transplantation. (B&C) Fluoromicrographs showed that MSCs engrafted (blue) in infarcted hearts underwent apoptosis (green) at 3 d and 1 w after transplantation, respectively(original magnification, 200×). (D) The percentage of TUNEL positive cells at 3 d, 1 w and 2 w after transplantation respectively. * indicates P < 0.05, the MSCs group 1 w after cell transplantation compared with 3 d after cell transplantation; # indicates P < 0.05, the MSCs group 2 w after cell transplantation compared with 1 w after cell transplantation. N = 3 for each group.

Numerous DAPI-labeled MSCs were found at the site of injection in MSCs group after transplantation (Figure 6A), which demonstrated successful cell engraftment. But the number of the DAPI-labeled cells reduced gradually over time. The TUNEL analysis confirmed that MSCs engrafted in infarcted hearts underwent apoptosis gradually over time (Figure 6B–D). No DAPI positive cells were found in control group and MSCs group 4 weeks after cell delivery.

### Distribution of superparamagnetic iron oxide particles and macrophages

**Figure 7:**
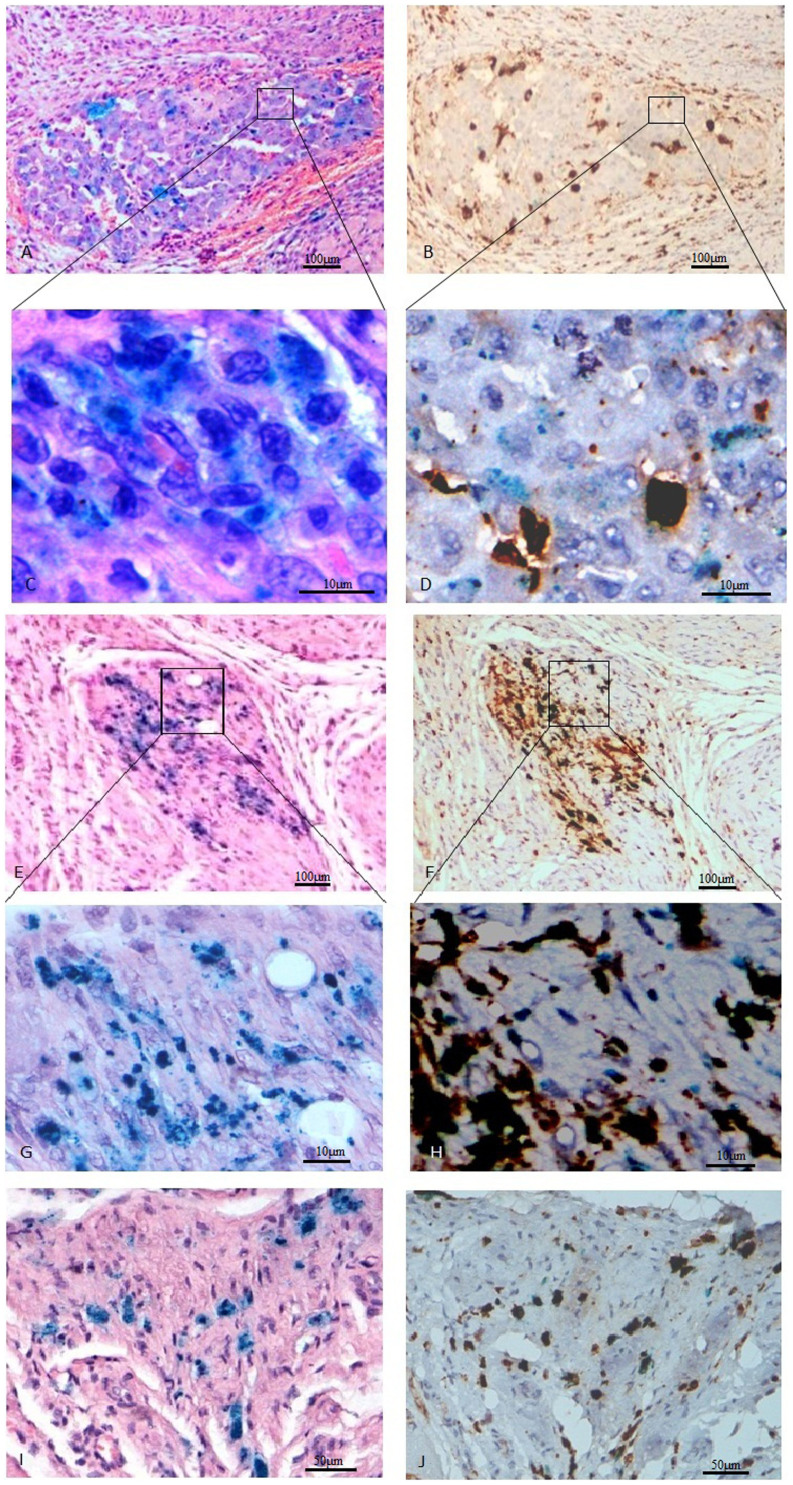
Photomicrographs of heart tissue sample at the site of MSCs injection. (A) Photomicrograph of costaining of (H&E) and iron 3 days after injection of SPIO-labeled MSCs (blue cytoplasm) showing clusters of iron-positive blue cells and confirmed cell engraftment (original magnification, 100×). (B) Colocalized photomicrograph of CD68 immunostaining(a marker of macrophages) and iron staining 3 days after cell transplantation(original magnification, 100×). (C) High-power magnification of iron stained cells(original magnification, 400×). (D) High-power magnification of CD68 immunostained cells(original magnification, 400×). (E) Photomicrograph of costaining of (H&E) and iron 1 week after injection of SPIO-labeled MSCs (original magnification, 100×). (F) Colocalized photomicrograph of CD68 and iron staining 1 week after cell transplantation(original magnification, 100×). (G) High-power magnification of iron stained cells(original magnification, 400×). (H) High-power magnification of CD68 immunostained cells(original magnification, 400×). (I) Photomicrograph of costaining of (H&E) and iron 2 weeks after injection of SPIO-labeled MSCs(original magnification, 200×). (J) Colocalization shows that many iron-positive cells are CD68-positive macrophages(brown cytoplasm) (original magnification, 200×).

The histopathological studies revealed that clustered blue particles were present in the site of cell injection (figure 7A,C,E,G,I). However, the results of CD68 staining showed that the infiltrated inflammatory cells increased over time and the distribution of most iron-positive cells were almost the same with the CD68-positive macrophages at 2 weeks after MSC transplantation (figure 7B,D,F,H,J), which indicated that most of the iron oxide nanoparticles were engulfed by macrophages.

### Detection of donor cell by real-time PCR for the SRY gene

**Figure 8:**
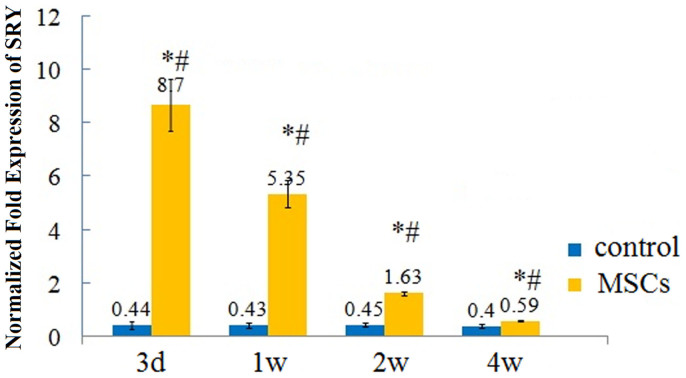
Real-time PCR analysis for the rat SRY gene. * indicates P < 0.05, the MSCs group compared with the control group at each time point; # indicates P < 0.05, the MSCs group 1 w, 2 w, 4 w after cell transplantation compared with 3 d after cell transplantation. N = 4 for each group.

For identifying the survival of transplanted cells of male donor origin, real-time PCR analysis for the rat Y-chromosome-specific SRY gene was performed from infarcted hearts of 4 female recipients in both groups at each time point. As shown in figure 8, the qPCR analysis revealed that the expressions of SRY-specific genes were remarkably decreased over time in MSC transplant group (8.70 ± 0.97 vs. 5.35 ± 0.63 vs. 1.63 ± 0.07 vs. 0.59 ± 0.03 for MSCs group at 3 days, 1 w, 2 w, 4 w after injection respectively) and no implanted male cells were detected in the control group. Moreover, the presence of engrafted cells was confirmed by the test on postmortem specimens, which showed that the amount of Y-chromosome-specific SRY gene of MSCs from male donors in infarcted hearts of female recipients was consistent with the results of the TUNEL assessment.

## Discussion

Transplantation of MSCs represents a promising therapy for MI, due to the proliferation and differentiation capacity of these cells. Understanding the fate of injected MSCs plays a critical role not only in the evaluation of the recovery of cardiac function but also in providing important insights into the mechanism of action of stem cells. Prior studies have reported on various in vivo imaging modalities to evaluate changes in cardiac structure and function used either in animal or clinical studies, including ultrasound, CT, single photon emission computed tomography scan (SPECT) and positron emission tomography scan (PET) and MRI. Because of its versatility, accuracy and reproducibility, MRI is currently considered a standard tool to evaluate the cardiac structure and function. Smits et al. measured the cardiac function after cardiac progenitor cell transplantation using magnetic resonance imaging and reported a higher ejection fraction and reduced extent of left ventricular remodeling^21^. Ziebart et al. also used MRI to assess the proangiogenic cells efficacy on cardiac function after transplanted into mice ischemic myocardium^22^.

In the present study, MRI was performed to assess the effectiveness of the stem cell therapy in real time by measuring various indices of LV remodeling and cardiac performance during the course of the 4-week study. In our longitudinal study of cardiac structure and function, MSCs appeared to improve the left ventricular ejection fraction and reduced the left ventricular end-systolic volume compared with baseline and the control group. But there were no statistical differences (P > 0.05), which was similar to the result reported in previous study^23^. Recent randomized controlled clinical trials using autologous bone marrow derived stem cells have failed to show significant long-term left ventricular functional improvement in MI patients^8^,^24^. The finding that MSCs transplantation resulted in little change in cardiac performance was not entirely unexpected, which was consistent with the post-mortem tissue histology showing the engrafted cells in infarcted hearts underwent necrosis or apoptosis gradually over time. These findings also suggested that the therapeutic effect of mesenchymal stem cells may come mainly from the stimulation of angiogenesis paracrine and activation of endogenous cardiac stem cells, rather than the transdifferentiation. The critical issue limiting the efficacy of stem cell transplantation is the short survival time of engrafted stem cells in the damaged heart tissue. However, how long the stem cells survived in vivo and the number of long-term survived cells have not been determined. Due to the various animal model used, transplantation method and location, the number of transplanted cells, the labeling and tracking technique, the results were different. Most studies limited to 3–8 weeks^12^,^13^,^14^,^15^,^25^. Also, there were some researches suggesting that the transplanted stem cells survived for more than 6 months in the scar tissue^3^,^26^. In this experiment, we found that little or no stem cells survived at 4 weeks after transplantation. Thus, improving the microenvironment of transplanted cells to enhance their survival and extend the survival time, may play a role in maintaining therapeutic effects. In addition, we did not find the existence of labeled MSCs in surrounding tissue of transplantation points. Therefore, it was inferred that the engrafted stem cells could not migrate, and the therapeutic effect may only act within the range of the graft site and its surrounding.

Understanding the fate of injected MSCs would provide important insights into the mechanism of action of stem cells. Although highly specific and sensitive, those histological and immunohistochemical methods can only provide information of implanted cells at the time of sacrificing the animal. The fate of implanted cells such as temporal changes of cell location, viability and functional status in clinical studies cannot be provided by invasive methods. Such information could be correlated potentially with objective clinical outcomes. The ability to quantify non-invasively the amount of survived MSCs and migration is of great clinical importance. SPIO were used as a kind of magnetic resonance contrast agent because of its paramagnetic characteristics that showed significantly negative signal in T2WI and T2*WI images. Recently, a number of studies used MRI to monitor the transplantation of SPIO-labeled cells in vivo. Küstermann et al. pointed out that the T2* WI imaging is most sensitive to SPIO-labeled stem cells, but its susceptibility effect to reduce signals could increase the area of low signal^27^. It was pointed out that under conditions of high field strength, SPIO labeling method can detect the presence of a single stem cell in vivo^28^. Here, we used a 7.0 T ultra-high-field-strength magnetic resonance imaging system and T2* W sequence to evaluate the implanted SPIO-labeled cells. SPIO do not directly image by itself, but through the influence with the longitudinal relaxation and transverse relaxation of the surrounding protons. SPIO produce disorder of the local magnetic field by increasing the magnetic intensity, lead to rapid loss of phase of protons around, result in signal changes detected by MRI. In this study,we injected MSCs into 35 rats hearts, and low signal areas were detected by MRI only in 31, which may be due to the failed transplantation. And we also found a reduction of signal contrast and area of low signal over time after cell transplantation. Kraitchman et al. showed a 24% reduction in 24 h, and pointed out that the possible causes include MSCs migrated and SPIO were cleared by the immune system after MSCs with SPIO split gradually^29^. It is known that iron particles may be phagocytosed by macrophages as cells died and decomposed after the labeled cells transplanted into the body, which interfered the magnetic resonance imaging to detect the transplanted cells. Several studies questioned the reliability of SPIO tracking the transplanted stem cells. Amsalem et al. transplanted the bone marrow mesenchymal stem cell in rats of myocardial infarction and found that SPIO remained in macrophages in the local myocardial tissue after 4 weeks using macrophage-specific surface markers CD68^13^. Terrovitis et al. demonstrated that MRI can only identify the presence or absence of iron particles in local tissue,but cannot distinguish whether it remained within the transplanted stem cells^15^. However,Kraitchman et al. failed to detect the presence of macrophages in the study^29^. In the present study, we found that the iron particles distribution consistent with the macrophages through Prussian blue and immunohistochemical staining, and that the implanted MSCs underwent apoptosis until disappeared through TUNEL detection at 2 and 4 weeks after cell transplantation. SRY gene expression analysis was consistent with the TUNEL results. After MI macrophages were activated to remove non-viable cells. Due to the microenvironment in MI heart,apoptosis or necrosis occurs after cell transplantation in vivo. So it was inferred that SPIO were engulfed by macrophages gradually over time. However MRI could not identify the SPIO from the transplanted cells or macrophages. Therefore, we suggested that SPIO could not accurately reflect the transplanted cells viability in the hearts as well as the number of survived cells and track the long-term fate of labeled MSCs but localize the injection site in vivo.

In the experiment, it was clearly observed that MRI demonstrated the position of SPIO labeled transplanted cells in the body as low signal area, and that the low signal area and intensity changed dynamically. A potential reason is the different scan level due to the pulsation of the heart. It cannot be excluded in spite of the interpretation of cells migration, apoptosis and necrosis. With further research and development, novel contrast agents, mechanisms for labeling and MRI sequences may help to overcome these challenges.

## Conclusions

In this study, we demonstrated that SPIO labeling method could not reliably track the long-term fate of MSCs engraftment in the heart—as there was rarely initially successfully engrafted MSCs cells alive over time and the iron-positive cells were macrophages within the heart. However, SPIO can be used to track whether stem cells were successfully transplanted into the target area of interest organ and to localize the area of injection in vivo.

## Author Contributions

N.M. designed the experiments, cultured the cells, performed statistical analyses, and drafted the manuscript. H.C., M.L. and H.Z. performed the MRI and analyzed the MR images. Q.L., X.C. and G.Y. performed the histological analysis. L.Z., X.M. and Y.T. assisted with the animal operation and culture. S.Z. provided the conception of the study and revised the manuscript. All authors reviewed the manuscript.
